# Prevalence of neuromyths among psychology students: small differences to pre-service teachers

**DOI:** 10.3389/fpsyg.2023.1139911

**Published:** 2023-05-05

**Authors:** Verena Novak-Geiger

**Affiliations:** School of Education, University of Klagenfurt, Klagenfurt, Austria

**Keywords:** neuromyths, psychology, teacher-training, SDT, prevalence, discrimination, response bias, misconceptions

## Abstract

Neuroscience will possibly aid the educational practice but neuromyths are prevalent worldwide. Certain misconceptions about learning, memory and the brain are prevalent in different groups and hard to dispel. Bridging the gap might be too far. However, Psychology may serve as a bridge between these distant fields. The present study examined neuromyth endorsement in psychology students. An online questionnaire based on 20 neuromyths and 20 neurofacts was used. Additionally, neuroscience exposure at university and media exposure was assessed. The sample consisted of psychology students (*N* = 116) in Austria and was compared to a teacher-training sample. The different groups were compared using Signal Detection Theory, Chi-square test, non-parametric correlation analyses, and independent sample *t*-test. No correlation between neuroscience exposure at university and leisure time for psychology students at the beginning of their studies could be found. Here, the same misconceptions were among the most prevalent—compared to the teacher-training students sample. Results show significant difference between the groups on discrimination ability and response bias. Although psychology students share the same most prevalent misconceptions, they differ significantly in their amount of agreement. The reported study reveals a better discernment ability and lower response bias on neuromyths in the Psychology students’ sample. On the individual item level, they performed better at rejecting some neuromyths than pre-service teachers. In conclusion, some neuroscience and pedagogical psychology training improves the ability to discriminate between true and false statements. Therefore, directly addressing these misconceptions within the study program—Teacher Training and Psychology—could reduce neuromyth endorsement.

## 1. Introduction

### 1.1. Neuromyths

As early as 2010, Neuroscience and Education have been announced as “An Ideal Partnership for producing Evidence-Based Solutions to Guide 21^st^ Century Learning” (Carew and Magsamen, 2010, p. 685) and neuromyths (NM) as possible barriers. NM are defined as misconceptions about the human brain, learning, and memory processes (OECD, Organisation for Economic Co-operation and Development, 2007, p. 107–125) and have been investigated intensively over the last years and are widely believed within the educational field in Europe (Dekker et al., 2012; Grospietsch and Mayer, 2019; Krammer et al., 2019), the United States (Macdonald et al., 2017; van Dijk and Lane, 2020), Canada (Blanchette Sarrasin et al., 2019), Latin America (Gleichgerrcht et al., 2015), China (Zhang et al., 2019) and other countries (Janati Idrissi et al., 2020). The most prevalent and persistent misconceptions are (1) that individuals learn better when they receive information in their preferred learning style (Learning Styles), (2) that the absence of exposure to a rich learning environment by the age of 3 leads to a loss of learning capacities (Importance of 3 Years), and (3) that differences in hemispheric dominance can explain individual differences among learners (Hemispheric Dominance; Torrijos-Muelas et al., 2020). Although some claim that misconceptions are not relevant to their teaching practice, for example for award-winning teachers (Horvath et al., 2018) or university student’s grades in the teacher training program (Krammer et al., 2021), others argue that they will “have serious consequences in the quality of education, as these beliefs pave the way for ill-grounded methodologies”(Ferrero et al., 2020, p. 2).

Different attempts have been made to explain the sources of neuromyths in recent years. For example, mass media (Zhang et al., 2019), outdated knowledge, and false interpretations with a kernel of truth (Grospietsch and Mayer, 2019) are responsible for the appearance of misconceptions. Additionally, certain cognitive biases, i.e.: confirmation bias—seem to be related to the belief in neuromyths (Rubin et al., 2022). For example, teachers believing in learning styles theory tend to discern and remember classroom situations as evidence that supports their view that learning information according to the individual preferred learning style aids understanding and remembering. Moreover, van Elk (2019) showed a relationship between neuromyths and a simple understanding of neuroscientific knowledge, a high need for cognitive closure, a fixed mindset, intuitive thinking, and in reverse scientific literacy. Considering science as static and unchanging and a need for unambiguous information predicted the belief in neuromyths. In other words, people who tend to form opinions relying on little information tend to believe in neuromyths. Similarly, people who rely on their intuition are expected to believe in neuromyths.

In addition to explaining the origin of neuromyths, several interventions to refute misconceptions have been investigated (Im et al., 2018; Lithander et al., 2021; Swire-Thompson et al., 2021) and different strategies have been used. On the one hand, no improvement in neuromyth belief could be found when taking a course in educational psychology (Im et al., 2018). Similarly, Macdonald et al. (2017) displayed that training in education or neuroscience results in a decrease but not a removal of false beliefs indicating that the gap between neuroscience and education might be too far. On the other hand, correcting neuromyths with refutation tasks was successful, also in the long term (Lithander et al., 2021).

To summarize, misconceptions about learning, memory, and the brain still exist in schools, among preservice and in-service teachers as well as headmasters. The knowledge about what practices deduced from notions of and statements about the brain are myths has not fully arrived in the educational setting yet. The high prevalence of neuromyths in the educational setting are a sign that the gap between neuroscience and education is wide and bridging this gap needs more interdisciplinary research (Thomas, 2019) and psychology could well aid as a link between neuroscience and education (Marsh et al., 2015; Wilcox et al., 2020) for two possible reasons. First, most psychology curricula include basic training in neuroscience and neurosciences are connected to topics covered in other psychological fields such as cognitive, social, or clinical neuroscience. Second, psychology alumni and alumnae are employed in different fields, as psychology is a multifaceted field. Students finishing their studies are engaged in clinical psychology, childcare, welfare institutions and as school psychologists—the belief in neuromyths may impede their professional practice. However, to the author’s knowledge, no psychologists or students of psychology have been investigated on their neuromyth prevalence so far.

### 1.2. Signal detection theory

Answering a questionnaire on statements about learning, memory, and neuroscience with a right/wrong response scheme, forces participants to make a decision. Several aspects could affect humans’ decisions (Grant et al., 2017). Hence, decisions are influenced by “(a) the prevalence of the characters[items] in the environment, (b) the expertise of the raters in detecting the characteristic, (c) the extent and direction of bias in their judgments, and (d) fluctuating levels of attention to the task (see Goldstein and Hogarth, 1997, […])” (Grant et al., 2017, p. 3). These influence the judgments’ reliability. For example, there will be more rare or common facts or myths the participant encounters (prevalence), and the participant’s expertise may vary (expertise), especially between different interest groups or professions. Furthermore, the participant’s bias and whether the questionnaire is answered with or without distraction, in the morning or late in the evening (attention level) will influence the decision. The Hit Rate and the False Alarm Rate are included within these measures and both are affected by expertise and bias. SDT (Green and Swets, 1966) attempts to separate “a rater’s ability from his or her response bias by defining a measure that reflects the *difference* between *Hit* and *False-Alarm Rates*” (Grant et al., 2017, p. 4). Accordingly, d’ is the ability to differentiate between truth and absence thereof.

In SDT, stimuli presented as targets and correctly identified as such, are referred to as “Hits” and targets not identified as such as “Misses.” Contrary, stimuli not presented as distractors and that are wrongly classified as targets are “False Alarms” whereas distractors not classified as targets are referred to as “Correct Rejections.” For example, Pennycook and Rand (2021), applied SDT to individuals falling for fake news. Here, truth discernment is the degree of believed misinformation in relation to correct information and was calculated similarly to sensitivity (d′) in SDT: “belief in true news” minus “belief in false news.” In their study, poor truth discernment relates to a deficit in careful reasoning, related knowledge, and the use of heuristics (familiarity and source). Similarly, SDT will be applied here, to assess how well participants can detect myths and facts in the current study. Therefore, the endorsement of neuromyths will be tested in a sample of psychology students, and the results will be compared to a sample of teacher-training students from a previous study (Krammer et al., 2019). The endorsement of myths and facts was defined as false alarms and hits, respectively. Similarly, denial of myths or facts was defined as correct rejections or misses. The SDT approach allows disentangling the ability to distinguish between true and false neuroscientific statements from a general response bias.

### 1.3. Hypothesis

Since information taught on neuroscience decreases but does not eliminate false beliefs about neuroscience, learning, and the brain (Macdonald et al., 2017; Rousseau, 2021) psychology students are expected to belief in neuromyths but show less neuromyth endorsement compared to teacher training students in Austria. Moreover, a difference in discrimination is expected because d’ entails neuromyth endorsement represented by false alarms. Previous studies including participants with higher exposure to neuroscience revealed only small differences between the public and teachers (Macdonald et al., 2017) but did not use Signal Detection Theory. In their study, Macdonald et al. (2017) referred to people with many completed university or college courses related to the brain and neuroscience as a high-exposure group. Similarly, psychology students are exposed to neuroscience in university courses. Here, the teacher-training curriculum includes an introductory course to teaching and learning with a small amount of educational psychology. In this course, among the characteristics of the pedagogical profession, also educational science, psychological and sociological foundations of teaching and learning in relation to pedagogical fields of action are taught (Curriculum, 2019). Therefore, compared to psychology students this knowledge is introductory and not in-depth knowledge of the topic.

## 2. Materials and method

In order to answer the hypothesis on neuromyth belief in psychology students and their differences to teacher training students, a quantitative online survey was used.

The initial sample consisted of 120 mostly undergraduate psychology students. Four of them had to be excluded because of missing data after demographics. The mean age was 22.27 years (*SD* = 4.77) and, the vast majority were female students (*N* = 83), one-third male (*N* = 32), and one person “divers.” All remaining participants were Bachelor Students of Psychology, with 81% in their first semester (*N* = 95). Most of the participants had A levels as their highest acquired educational degree (*N* = 98) whereas some already hold a bachelor’s degree (*N* = 13). For a comparison of the present sample of psychology students with students in the educational field, the data from Krammer et al. (2019), made available at: https://osf.io/5tsfv/ (Krammer et al., 2019), was used with the permission of the first author. Here, 24 participants were excluded from the analysis due to missing data on age or semester, and a great number of missing values. Then, the final sample consisted of 648 students with a mean age of 20 (*SD* = 3), the vast majority in their first semester (*N* = 613) and being female participants (*N* = 416) compared to male participants (*N* = 233). Within this sample, no data on proficiency in neuroscience or exposure to neuroscience was collected.

The questionnaire used to investigate neuromyth endorsement was based on Dekker et al. (2012) and Krammer et al. (2019) containing 20 neuromyths and 20 neurofacts, demographic data, and neuroscience exposure either at university or in private were used. Participants were asked to report on attended lectures that included neuroscience or the brain as topics; on lectures or seminars primarily on neuroscientific topics and/or the brain; whether they attended related undergraduate introductory lectures and/or more advanced level courses within the curriculum. Moreover, they were questioned on their leisure time spent on topics such as brain and neuroscience and learning and memory. Additionally, two items were used for participants’ self-rating on a 5-point scale (very bad, bad, medium, good, very good) for their neuro-knowledge and knowledge about learning and memory. In more detail, two questions to assess neuroscience exposure at university were included in the questionnaire: *Are or were the topics “neuroscience” or “brain” components of courses at the AAU that you attended?* and *Have you already taken one or more courses on the topic of learning?* The first employs a yes/no response option, and the second a three-point scale (none, one, several). Moreover, included were yes/no questions on the attendance of the introductory lecture and more advanced lecture in neuroscience (*Have you already attended the lecture Cognitive Neuroscience A?* and *Have you already attended the lecture Cognitive Neuroscience B?*) and questions on neuroscience exposure outside the university. One general question *(Do you spend your free time on topics related to the brain or neuroscience?)* and two questions on media exposure on a 4-point Likert Scale (never, seldom, sometimes often) were used (*Do you regularly watch shows on TV or streaming platforms that focus on neuroscience topics and the brain?; Do you regularly watch shows on TV or streaming platforms that focus on topics learning and memory?*).

The questionnaire was an online study using LimeSurvey Software and was sent to psychology students enrolled in a lecture at the end of their first semester via email by the lecturer. The attendees were able to participate in the waffling of a voucher and partial course credit.

Data was analyzed with IBM SPSS Statistics 28.0.0.0. Chi-square test for the effect sizes presented and Cramer’s V (small effect ≤ 0.08; moderate effect ≤ 0.22; large effect ≥ 0.35) was used. Previous studies examining the underlying factor structure of neuromyths and neurofacts could not find a common factor for neuromyth items (Macdonald et al., 2017; Horvath et al., 2018; Krammer et al., 2019).

This study was carried out in accordance with the recommendations of the Institutional Review Board of the University of Klagenfurt with informed online consent from all subjects. The University of Klagenfurt Ethics Committee approved the study protocol. The ethics approval, study material, raw data and script is openly available at: https://osf.io/ndzwp/.

## 3. Results

The results section is structured as follows. First, I report neuroscience expertise in the psychology sample and the relationship with measured demographic variables and neuromyth acceptance and denial (frequencies, descriptive analyses, Spearman correlation) to answer whether neuroscience knowledge correlates with neuromyths denial and neuromyths acceptance. Next, I compare psychology students and teacher training students on the item level of the questionnaire (Proportions, Chi-square test, Cramer’s V) to answer whether these groups differ in their neuromyth endorsement. Finally, I describe the results of the comparison of discrimination ability and response bias of and between the groups (SDT analysis, independent sample *t*-test) to compare psychology and teacher-training students.

### 3.1. Neuroscience expertise

Descriptive data analysis indicate that psychology students are not immune to misconceptions about learning and the brain although exposed to neuroscience through lectures and leisure activities. Among psychology students, 95% (*N* = 110) stated, that neuroscience or the brain were topics in courses they attended so far, and 87% (*N* = 101) attended an introductory lecture but only 7% (*N* = 9) attended an advanced lecture on cognitive neuroscience. Regarding neuroscience exposure outside university, 46% (*N* = 53) were not concerned with this topic in their free time compared to 53% (*N* = 62) with some exposure, and 1% (*N* = 1) reported high exposure. Within the psychology students sample, media exposure related to neuroscience and the brain was 31% (*N* = 36) never, 56% (*N* = 65) seldom, and 12% (*N* = 14) sometimes and 1% (*N* = 1) often. Additionally, media exposure related to learning and memory was 32% (*N* = 37) never, 49% (*N* = 57) seldom, 17% (*N* = 20) sometimes and 2% (*N* = 2) often. Participants’ self-rating for their neuro-knowledge was 5% (*N* = 6) very bad, 47% (*N* = 54) bad, 45% (*N* = 52) medium and 3% (*N* = 4) good. Participants’ self-rating of their knowledge about learning and memory was 2% (*N* = 2) very bad, 31% (*N* = 36) bad, 56% (*N* = 65) medium and 11% (*N* = 13) as good.

The prevalence of misconceptions about learning and the brain in the psychology student sample compares to previous studies with teachers, headmasters, and teacher training students’ samples. Table 1 displays the response proportions (percentage) and statistics for each item. Among the psychology students sample the highest false alarms are seen with *NM9 “Students learn better when information is presented according to their learning type”* (91%) followed by *NM15 “Short-term coordination exercises help to better integrate the left and right hemisphere*” with 67 percent wrong agreement with the statement. The third was 64% *NM18 Lessons should be designed in such a way that both sides of the brain are addressed*. The highest proportion of correct rejections (Neuromyths classified as “wrong”) received items *NM16* “*The brain is not active when we sleep.”* (97%), and Item *NM13* “*Intelligence is inherited and not changeable by the environment”* (85%). Almost half of the participants (47%) were uncertain about the classification and chose “do not know” as an answer on *NM3* “*It is scientifically proven that fatty acid (omega-2, omega-6) containing food supplements have a positive effect on academic success.*” as well as on item *NM12* “*Body-eye coordination exercises can positively affect reading ability.*” (41%) and *NM10* “*Sensory-rich environments improve brain development in kindergarten children.”* (37%).

**Table 1:** Spearman correlations among measured variables in psychology student’s sample.

	1	2	3	4	5	6	7	8	9	10	11	12	13	14
1. NM consent	–													
2. NM rejection	**−0.219**	–												
3. NF consent	**0.265** ^**^	0.043	–											
4. NF rejection	0.116	**0.556** ^**^	**−0.272** ^*^	–										
5. Discrimination (d′)	**−0.575** ^**^	0.011	**0.365** ^**^	**−0.726** ^**^	–									
6. Response bias (c)	**0.438** ^**^	0.718^**^	**−0.408** ^**^	**−0.794** ^**^	−0.278	–								
7. Lecture A	0.079	0.094	−0.008	−0.078	0.033	−0.066	–							
8. Lecture B	0.062	−0.046	−0.135	−0.004	−0.072	0.051	0.126	–						
9. Course Topic	0.162	−0.051	−0.157	−0.065	−0.103	−0.095	**0.491** ^**^	0.076	–					
10. TV neuro	0.056	0.029	−0.080	0.124	−0.180	0.103	−0.159	0.076	0.136	–				
11. TV memory	0.077	0.028	−0.115	0.119	−0.177	0.068	−0.025	0.005	0.204	**0.540** ^**^	–			
12. Self-rating neuro	0.081	0.050	0.017	0.089	−0.115	0.084	**−0.208**	−0.092	0.117	**0.270** ^*^	0.184	–		
13. Self-rating memory	0.174	0.016	0.028	−0.009	−0.063	0.047	0.105	−0.156	**0.299** ^**^	0.263^*^	0.163	**0.471** ^**^	–	
14. Leisure	0.123	−0.0	−0.004	−0.029	−0.086	0.045	0.003	0.074	0.062	**0.385** ^**^	**0.318** ^**^	**0.264**	0.166	–

Significant correlations are printed in bold; ^*^p ≤ 0.005; ^**^p ≤ 0.001.

The distributions of the frequencies for neuromyth denial, neuromyth acceptance, neurofacts denial and neurofacts acceptance were not normal as indicated by Kolmogorov–Smirnov and Shapiro–Wilk tests (*p* < 0.001 and *p* > = 0.052). As a result, non-parametric Spearman Correlations were used.

Spearman correlation analysis on demographic variables in the psychology students’ sub-sample were computed and are displayed in Table 1. No significant correlation between neuromyth consent and demographic variables as well as neurofact consent and demographic variables could be found. However, neuromyth consent showed a small significant correlation with neurofact consent (*r* = 0.26, *p* < 0.005), a medium negative significant correlation with d prime (*r* = −0.575, *p* < 0.001), a medium negative correlation with response bias c (*r* = −0.438, *p* < 0.001). Neuromyth rejection showed medium significant correlation with neurofact rejection (*r* = 0.556, *p* < 0.001). Neuromyth rejection showed a high correlation with response bias (*r* = 0.718, *p* < 0.001). Additionally, neurofact consent (correctly accepting neurofacts) showed a small negative correlation with neurofact rejection (*r* = −0.272, *p* < 0.005), a small correlation with neuromyth consent (*r* = 0.265, *p* < 0.005) as well as a small negative correlation with neurofact rejection (*r* = −0.272, *p* < 0.005). Moreover, neurofact rejection showed a medium correlation with neuromyth denial (*r* = 0.556, *p* < 0.001) and a small negative correlation with neurofact consent (*r* = −0.272, *p* < 0.005).

Participants’ self-rating on their neuroscientific knowledge showed a small significant correlation with *leisure* (*r* = 0.26, *p* = 0.004), and a medium significant correlation with their self-rated knowledge on memory and learning (*r* = 0.47, *p* < 0.001). Additionally, a small correlation between self-rated neuro-knowledge and watching broadcasts or documentaries depicting neuroscientific topics (*r* = 0.27, *p* = 0.003). Participants’ self-rating on their knowledge on memory and learning showed the higher the self-rated knowledge on the topic memory and learning, the more courses that depicted the topics were attended (*r* = 0.30, *p* < 0.001).

### 3.2. Item-level comparison for psychology students and teacher-training students

The teacher training students sample shares the first three most prevalent misconceptions in place with the psychology students sample: *NM9* “*Students learn better, when information is presented according to their learning type Coordination exercises help to better integrate hemispheres”* (97%), *NM15* “*Short-term coordination exercises help to better integrate the left and right hemisphere”* (88%) and *NM18 “Lessons should be designed in such a way that both sides of the brain are addressed.*” (86%) were the most prevalent Neuromyths among pre-service teachers. Moreover, *NM5 “Brain dominance (left/right) explains individual learning differences.”* (82%) and *NM12 “Body-eye coordination exercises can positively affect reading ability.”*(80%) were believed by the vast majority of the teacher training sample. Similar to the psychology students. The highest proportions of correct rejections of neuromyth items among the teacher-training sample received item *NM16 “The brain is not active when we sleep.* “(95%).

A 2 (background: psychology, teacher-training) × 3 (response to item: correct, incorrect, do not know) Chi-square analysis (*df* = 2) on the responses of the neuromyth items between psychology students and teacher training students is displayed in Table 2 and revealed that 15 of the 20 items differed significantly in their response patterns. The effect sizes of five neuromyth items were small (*NM 11*, *NM, 13*, *NM16*, *NM 19*, and *NM 20*) whereas the effect sizes for the remaining 15 items were moderate. The highest effect size could be found for four items *NM4*, *NM5*, and *NM12* and *NM18*. Psychology students were less likely to agree on the neuromyth *NM5* that “*Brain dominance (left/right) explains individual learning differences*” compared to teacher training students (54 vs. 82%, effect size Cramer’s V 0.304) as well as on neuromyth *NM4* “*We only use 10% of our brain*” (15 vs. 44%, effect size Cramer’s V 0.285). Moreover, neuromyth *NM12 “*Body-eye coordination exercises can positively affect reading ability.” was less believed by psychology students (41%) compared to teacher training students (80%) with an effect size of Cramer’s V 0.275. Here, psychology students used the “do not know” category more often (41%) compared to teacher training students (16%). *NM18* “*Lessons should be designed in such a way that both sides of the brain are addressed”* was less believed in the psychology students sample compared to teacher training students (64 vs. 86%, effect size Cramer’s V 0.244). Additionally, Psychology students were better at rejecting *NM15* “*Short-term coordination exercises help to better integrate the left and right hemisphere*” compared to teacher training students (67 vs. 88%, effect size Cramer’s V 0.222). However, psychology students chose “do not know” more often than teacher-training students.

**Table 2:** Percentage of responses between psychology students and teacher training students on each neuromyth item, together with item-level Chi2 test statistics (all *df* = 2).

Neuromyths	Psychology students			Teacher training students					
	R	W	DK	R	W	DK	Chi^2^	*p*	Cramer’s *V*
(NM1) The first language must be acquired before the second language is acquired completely.	19(23)	63(71)	18(22)	35(233)	54(361)	11(77)	12.15	**0.002**	0.124
(NM2) When students do not drink enough water (6–8 glasses), their brains shrink.	13(13)	63(73)	24(31)	5(32)	75(502)	20(137)	10.90	**0.001**	0.117
(NM3) It is scientifically proven that fatty acids (omega-2, omega-6) containing food supplements have a positive effect on academic success.	25(29)	28(33)	**47(54)**	38(255)	16(107)	**46(311)**	13.35	**0.001**	0.130
(NM4) We only use 10% of our brain.	15(17)	78(90)	8(9)	44(294)	38(253)	18(123)	63.89	**<0.001**	**0.285**
(NM5) Brain dominance (left/right) explains individual learning differences.	**54(63)**	20(23)	26(30)	**82(552)**	3(18)	15(101)	72.64	**<0.001**	0.**304**
(NM6) The brains of boys and girls develop at the same rate.	31(35)	45(53)	24(28)	19(125)	55(366)	26(175)	8.04	**0.018**	0.101
(NM7) Brain development is completed between the ages of 11 and 12.	3(4)	81(93)	16(19)	4(25)	67(446)	30(198)	8.896	0.**012**	0.106
(NM8) In childhood, there are critical phases, after which certain things can no longer be learned.	38(44)	43(50)	19(22)	51(340)	29(197)	20(135)	9.318	0.**009**	0.109
(NM9) Students learn better when information is presented according to their learning type.	**91(107)**	6(7)	3(3)	**97(654)**	1(6)	2(12)	16.343	**<0.001**	0.144
(NM10) Sensory-rich environments improve brain development in kindergarten children.	38(46)	25(27)	**37(44)**	57(383)	15(104)	28(186)	12.577	**0.002**	0.126
(NM11) Children are less receptive after consuming sugary snacks and/or drinks.	40(46)	27(31)	33(39)	39(262)	35(263)	26(174)	4.254	0.119	0.073
(NM12) Body-eye coordination exercises can positively affect reading ability.	47(54)	12(14)	**41(48)**	**80(536)**	4(24)	16(111)	59.624	**<0.001**	**0.275**
(NM13) Intelligence is inherited and not changeable by the environment.	10(11)	**85(98)**	5(6)	7(46)	**85(570)**	8(55)	2.118	0.347	0.052
(NM14) Learning difficulties related to developmental differences in brain function cannot be corrected by education.	33(39)	39(45)	28(33)	19(127)	47(319)	**34(225)**	12.457	**0.002**	0.126
(NM15) Short-term coordination exercises help to better integrate the left and right hemispheres.	**67(78)**	9(10)	24(29)	**88(593)**	4(29)	7(50)	36.010	**<0.001**	**0.222**
(NM16) The brain is not active when we sleep.	1(1)	**97(113)**	2(2)	1(9)	**95(673)**	4(24)	1.269	0.530	0.040
(NM17) There is not just one but several independent intelligences localized in different brain regions.	50(58)	19(22)	30(35)	61(408)	7(50)	**32(212)**	16.423	**<0.001**	0.145
(NM18) Lessons should be designed in such a way that both sides of the brain are addressed.	**64(75)**	14(16)	22(26)	**86(575)**	2(15)	12(82)	46.829	**<0.001**	**0.244**
(NM19) Going to school for several years makes children less creative. Children are most creative before entering school.	33(38)	35(41)	32(37)	43(287)	31(210)	26(174)	4.222	0.121	0.073
(NM20) Highly gifted people do not need to learn to perform well in school.	4(5)	**89(103)**	7(9)	5(33)	**87(585)**	8(53)	0.99	0.951	0.011

R, W, DK refer to the response codes right, wrong, do not know. Numbers printed in bold highlight similarities or differences between the group’s responses.

### 3.3. Discrimination ability and response bias

Next, the ability to discriminate myth and fact in neuroscience statements was tested between the two samples using SDT. Right (R) and wrong (W) answer categories were included, and do not know (DK) were excluded from the analysis. Hit and false alarm rates (endorsements of neurofacts versus neuromyths) were computed individually, as were discrimination ability d’ and response bias c (see Macmillan and Creelman, 2005). Bias c values of zero reflect unbiased, neutral responding. In the present setting, positive values of bias c reflect the conservative tendency to rather endorse statements as false and negative values reflect liberal responding and the tendency to endorse statements as true.

**Figure 1:**
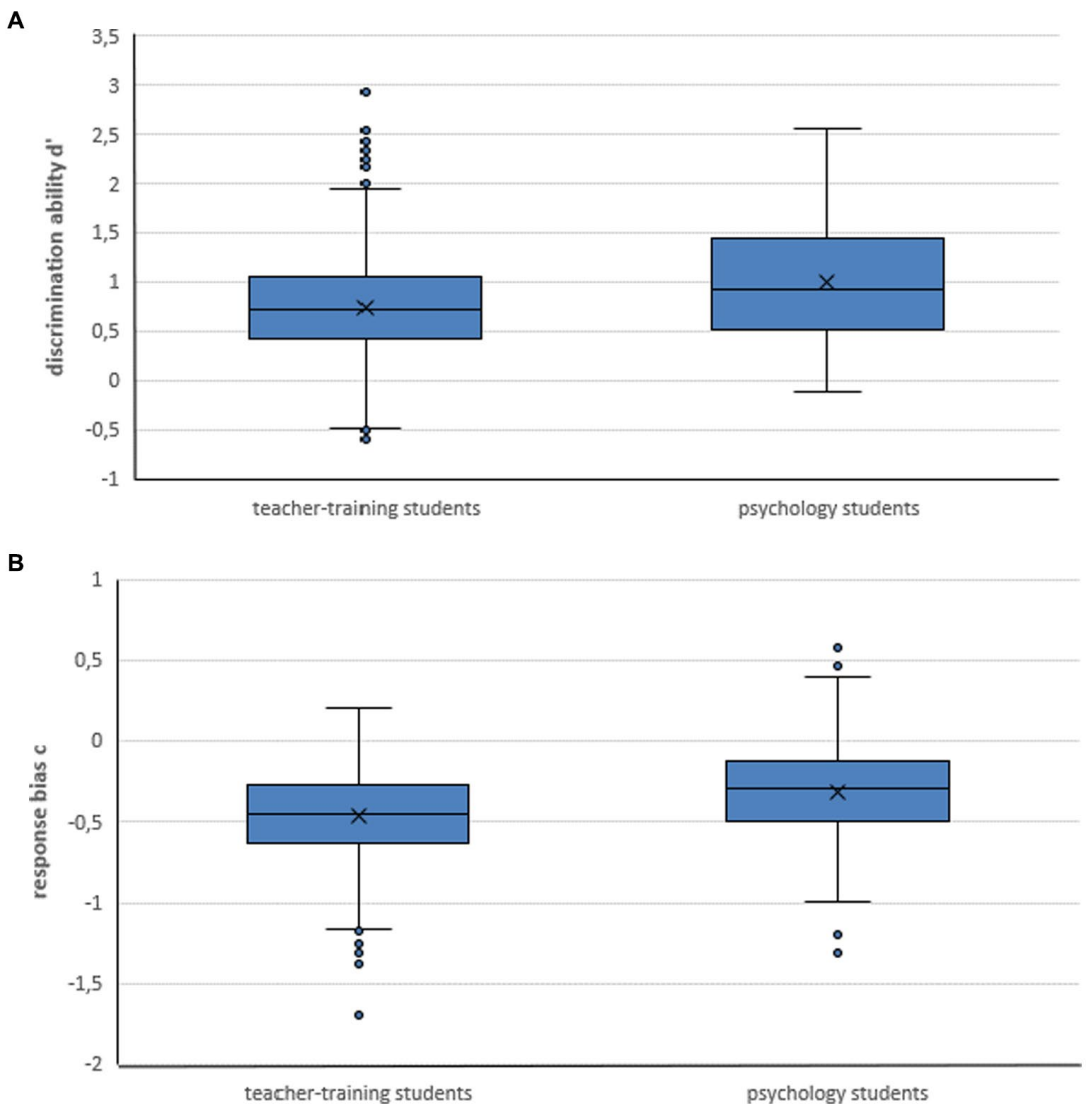
Boxplots of discrimination ability d’ **(A)** and response bias c **(B)** in the neuromyth questionnaire for the psychology and teacher training samples; crosses reflect means.

In the psychology sample, the mean hit rate was *M =* 12.1 (*SD* = 2.8), and the mean false alarm rate was *M =* 6.7 (SD = 2.6). The mean hit and false alarm rates in the teacher training sample were *M* = 12.3 (*SD* = 2.6) and *M* = 8.5 (*SD* = 2.2). Distributions of d′ and response bias c for both groups are shown in Figure 1. An independent samples *t*-test on the ability to discriminate (d′) and the response bias (c) was conducted on the two samples. Discrimination ability d’ was significantly higher for psychology than for teacher training students (*M* = 0.99, [*SD* = 0.57], *M* = 0.74 [*SD* = 0.49]; *t*(145.905) = −4.540, *p* < 0.001, *d* = 0.51). Similarly, response bias c was significantly smaller for psychology than for teacher training students (*M* = −0.31, [*SD* = 0.36], *M* = –46, [*SD* = 0.28]; *t*(139.322) = −0.437, *p* < 0.001, *d* = 0.53). Discrimination ability and response bias are displayed in boxplots in Figures 1A,B for both samples.

Moreover, correlations of variables with discrimination ability d’ and response bias c are shown in Table 1. Here, Neurofact consent showed a small correlation with discrimination ability d prime (*r* = 0.365, *p* < 0.001) and a similar but negative correlation with response bias (*r* = −0.408, *p* < 0.001). Neurofact rejection showed a high negative significant correlation with d prime (*r* = −0.726, *p* < 0.001) and response bias c (*r* = 0.794, *p* < 0.001).

## 4. Discussion

In the present study, descriptive data analysis indicate that psychology students are not immune to misconceptions about learning and the brain—though they are to some extent trained in neuroscience. Here, the most prevalent misconceptions were (1) *Learning styles*, (2) “*Short-term coordination exercises help to better integrate the left and the right hemisphere”* and (3) the notion that *“Lessons should be designed in such a way that both sides of the brain are addressed.”* In the Austrian pre-service teacher sample by Krammer et al. (2019), the same neuromyths showed the highest prevalence. These groups were then compared.

A difference on the individual item level on some questions could be shown. The largest significant difference was discovered for the item *“Brain dominance (left/right) explains individual learning differences*.”: Psychology students were less likely to accept this statement as correct. Similarly, they were more likely to identify “*We only use 10% of our brain*” as incorrect and did not accept “*Body eye coordination exercises can positively affect reading ability”* as often as teacher-training students as a true statement. The groups answered differently on “*Lessons should be designed in such a way, that both sides of the brain are addressed*.” and “*Short-term coordination exercises help to better integrate the left and right hemisphere.*” Again, psychology students were less likely to accept these statements as true. The group differences on individual items could have three different causes. First, the statements about brain dominance and on designed lessons addressing both sides of the brain can be attributed to the distinction in the student’s desired profession and the resulting study content. Prospective teachers are more concerned with learning and differentiated teaching than psychology students are. Psychology is the study of the human psyche and behavior. Second, psychology students used the answer category “do not know” more often for some of those items (*Body eye coordination exercises can positively affect reading ability; Short-term coordination exercises help to better integrate the left and right hemisphere*). Here, the lower self-rating of neuroscientific knowledge these students reported and/or the notion of the complexity of the human brain after attending the introductory lecture on cognitive neuroscience could be a possible cause. Third, the vast majority of the psychology students correctly classified the statement “*We only use 10% of our brain*” as wrong, compared to teacher training students. Again, more (introductory) knowledge on the brain may serve as a reason. For teacher-training students, addressing neuromyths in lectures and courses could improve the belief in neuromyths.

Psychology students’ self-rating on their neuroscientific knowledge showed a correlation with leisure—time spent on neuroscientific topics in the free time. Moreover, participants’ self-rating of their knowledge of memory and learning is connected to the amount of time spent watching documentaries and broadcasts related to learning, and memory. Participants self-rating on neuroscience and learning and memory are related as well. Attended introductory lecture on neuroscience and/or advanced lecture on neuroscience showed no correlation with neuromyth consent or denial and no correlation with deeprime discrimination ability and response bias. University courses depicting the topic of neuroscience, learning and memory showed a small correlation with participants’ self-rating memory and learning. They might feel more confident due to the gained knowledge. No significant correlation of demographic variables with neuromyth belief or denial, neurofact acceptance or denial was found. Here, the demographic However, this data was not available for the compared teacher-training students.

The initial hypothesis predicted that psychology students do not differ significantly in their prevalence of neuromyths to teacher-training students in Austria because the amount of neuroscience exposure is not sufficient to make a difference in the prevalence. Firstly, the survey responses depict a similar picture of the most prevalent statements in both samples. Although the same misconceptions are most prevalent in both samples, psychology students’ neuromyth acceptance (false alarms) differs significantly from the teacher-training students. Initial training in neuroscience and in topics related to learning and memory makes a difference in the percentage of neuromyth endorsement for individual items. Future teachers’ attention should be drawn to the complexity of the human brain and difficulty in formulating (simple) recommendations for lessons. Moreover, more knowledge about neuroscience would be a protective factor. Additionally, a SDT analysis revealed that both groups were similar in their percent of correct answers on neurofacts, and discrimination ability was different as well as response bias. Moreover, an independent sample *t*-test on these measures revealed significant difference between psychology students and pre-service teachers. Psychology students showed a higher discrimination ability and were therefore better at distinguishing between correct and incorrect statements.

However, the present study faces certain limitations. The items used in the questionnaire need development, as Sullivan et al. (2021) suggested. Some statements cannot be clearly classified into myth or fact because the evidence is ambiguous. Moreover, precise reading is essential to recognize the difference between fact and fiction in some items (for example, critical period vs. sensible period in childhood). Another improvement could be achieved in statements with more context information in contrast to one-sentence statements. Additionally, the teacher-training student sample does not contain demographics on neuroscience exposure, making a comparison difficult.

Within education, Learning Styles are not seen as a holistic concept. Confusion with theories of learning is sometimes understood as Visual–Auditory-Reading-Kinaesthtik (VARK; Fleming and Mills, 1992) framework, and sometimes as multiple intelligences by Gardner. Additionally, they find their entry into teaching via techniques (Papadatou-Pastou et al., 2021). Although instruction based on learning styles does not result in an improvement in learning (Rogowsky et al., 2020) the concept is still being used [for example in Çam et al. (2022)]. Future research could aim at examining the different understandings of learning styles, as this neuromyth received the highest amount of wrong answers. Similarly, psychology students may not use the same concept of learning styles. Here, qualitative research could be employed. These studies could use qualitative approaches or experimental approaches to gain a deeper understanding of people’s understanding of neuromyths, their knowledge, and their application. Qualitative research may address individual neuromyths, for example, the most prevalent misconception on learning styles. Additionally, future research may focus on the question of whether graduated psychology students have gained knowledge that protects against the belief in neuromyths, how this knowledge develops, and if the proportion of correct answers on the misconceptions and facts about learning, memory and the brain changes.

Furthermore, the dissemination of misconceptions in schools and at the tertiary level can be suspended even though knowledge in neuroscience not directly coincides with neuromyth denial. As recently shown by Ruiz-Martin et al. (2022), interventions with in-service teachers resulted in a reduction of Neuromyths. Moreover, addressing these misconceptions directly within the curriculum could result in an improvement. Therefore, strategies to encounter misinformation, as described by Ecker et al. (2022) could be used. “Intervention approaches that focus on both activating rational thinking (i.e., refutation-based interventions) and mitigating intuitive thinking, as well as non-prescriptive approaches like teacher professional development workshops and seminars on the neuroscience of learning, are promising avenues to dispel beliefs in neuromyths and to instill evidence-based teaching practices in the classroom, respectively.” (Rousseau, 2021, p. 9).

## Data availability statement

The datasets presented in this study can be found in online repositories. The names of the repository/repositories and accession number(s) can be found at: https://osf.io/ndzwp/.

## Ethics statement

The studies involving human participants were reviewed and approved by Institutional Review Board of the University of Klagenfurt. The participants provided their written informed consent to participate in this study.

## Author contributions

The author confirms being the sole contributor of this work and has approved it for publication.

## Funding

The study was partly funded by the School of Education, University of Klagenfurt, Klagenfurt, Austria.

## Conflict of interest

The author declares that the research was conducted in the absence of any commercial or financial relationships that could be construed as a potential conflict of interest.

## Publisher’s note

All claims expressed in this article are solely those of the authors and do not necessarily represent those of their affiliated organizations, or those of the publisher, the editors and the reviewers. Any product that may be evaluated in this article, or claim that may be made by its manufacturer, is not guaranteed or endorsed by the publisher.
